# In Situ 3D-Imaging of the Inner Ear Synapses with a Cochlear Implant

**DOI:** 10.3390/life11040301

**Published:** 2021-04-01

**Authors:** Kathrin Malfeld, Nina Armbrecht, Holger A. Volk, Thomas Lenarz, Verena Scheper

**Affiliations:** 1 Department of Otolaryngology, Hannover Medical School, Carl-Neuberg-Str. 1, 30625 Hannover, Germany; malfeld.kathrin@mh-hannover.de (K.M.); armbrecht.nina@mh-hannover.de (N.A.); lenarz.thomas@mh-hannover.de (T.L.); 2 Department of Small Animal Medicine and Surgery, University of Veterinary Medicine Hannover, 30559 Hannover, Germany; Holger.Volk@tiho-hannover.de; 3 Cluster of Excellence “Hearing4all”, German Research Foundation, DFG (Deutsche Forschungsgemeinschaft), Hannover Medical School, Carl-Neuberg-Str. 1, 30625 Hannover, Germany

**Keywords:** cleared cochlea, ribbon synapse, confocal laser scanning microscopy

## Abstract

In recent years sensorineural hearing loss was found to affect not exclusively, nor at first, the sensory cells of the inner ear. The sensory cells’ synapses and subsequent neurites are initially damaged. Auditory synaptopathies also play an important role in cochlear implant (CI) care, as they can lead to a loss of physiological hearing in patients with residual hearing. These auditory synaptopathies and in general the cascades of hearing pathologies have been in the focus of research in recent years with the aim to develop more targeted and individually tailored therapeutics. In the current study, a method to examine implanted inner ears of guinea pigs was developed to examine the synapse level. For this purpose, the cochlea is made transparent and scanned with the implant in situ using confocal laser scanning microscopy. Three different preparation methods were compared to enable both an overview image of the cochlea for assessing the CI position and images of the synapses on the same specimen. The best results were achieved by dissection of the bony capsule of the cochlea.

## 1. Introduction

Sensorineural hearing loss (SNHL) is primarily caused by the initial loss of hair cells in the Organ of Corti of the inner ear. Following hair cell loss the afferent fibers of the spiral ganglion neurons (SGN) degenerate and subsequently the SGN and their central projections die [1,2,3]. For decades, basic research on SNHL focused, among others, on elucidating the underlying pathologies at the level of the SGN and developing therapy strategies to ensure the survival of these neurons and outgrowth of their neurites. Meanwhile it is known that the pathological cascades leading to SNHL are not always following the canonic way. In some cases of SNHL the ribbon synapses between the inner hair cells and the peripheral neurites of the SGN are identified as being initially traumatized, resulting in a synaptopathy [4,5,6,7]. Noise damage may lead to hearing loss—without hair cell loss but with a severe synaptopathy. Sometimes there is no threshold shift but a reduction of the wave I amplitude in the auditory brainstem response [8]. This phenomenon is called “hidden hearing loss” and the affected patients struggle with hearing in noisy environments. Additionally the loss of ribbon synapses can also lead to temporary threshold shifts [9,10]. Besides noise a synaptopathy can also be caused by age [11] or genetic lesions. In humans there are four genes each known to cause presynaptic and postsynaptic synaptopathies [12].

Within the last years the ribbon synapses gained interest not only in basic research, to understand the function of normal hearing and the mechanisms of trauma, but also in the development of new strategies for the prevention and therapy of hearing loss. This includes advanced therapies for cochlear implant (CI) patients. Although the CI leads to a direct stimulation of the spiral ganglion neurons and this way circumvents the ribbon synapses, the analysis of ribbon synapses is important due to the fact that the CI outcome is, among others, dependent on the preservation of the residual hearing [13] and therefore on healthy ribbon synapses of the intact hair cells.

For research purposes the cochlear implanted inner ears have to be examined histologically. This is a challenging procedure due to its complex structure, the three fluid-filled spaces, and the variety of cell types ranging from thin membranes to compact lamellar bone. Due to the compact lamellar bone, the densest in mammals [14], forming the otic capsule it is impossible to image the organ of Corti, which is the sensorineural organ of special interest, through the bony capsule. Therefore the common way is to separate the neuroepithelium [15,16], or to embed the tissue in various materials, e.g., paraffin [17,18], plastic [19,20] including resin [21,22], or gelatin [23] and produce serial sections or grind it for histological evaluation. The resulting 3D reconstructions are time-consuming procedures and are prone to artefacts [19,24]. In addition, embedding materials can result in less tissue antigenicity, making it difficult to perform immunostaining. If paraffin or gelatin embedded implanted cochleae shall be sectioned, the electrode array of the CI, consisting, among others, of platinum, has to be removed. This makes it difficult and sometimes impossible to evaluate the basic information like the array position in the inner ear or the amount of fibrosis around the implant. Plastic embedded samples can be sectioned without removing the implant but the implant-material will get lost from the section since the implant is not attached to the embedding material. In addition, due to the cutting procedure, hard plastic cuts lead to a high loss of tissue material, which prevents small-scale distance analyses. Another option for histological assessment is the grinding of plastic embedded specimens, which leads to a complete loss of the tissue and does allow only limited immunocytochemistry. To circumvent these limitations a clearing protocol for cochleae was developed and became popular in the last years. It is now used in mice [25], gerbils [26,27], and guinea pigs [28,29,30] for whole cochlea imaging with confocal laser scanning microscopy (CLSM). Cleared cochleae enable the evaluation of intact tissue without sectioning. The substance used for clearing in these studies is called Spalteholz solution which allows leaving a CI in situ during histological evaluation to allow the detection of the electrode position and to quantify the fibrosis around the implant [30]. Furthermore, it has been shown, that immunostaining of hair cells, neurons [25,27,31], and fibrosis [28,30] of the cleared specimens is possible. Another clearing method is iDISCO+, which is used for the clearing of rat temporal bones [32]. To our knowledge no staining of hair cell synapses of cochlear implanted specimens has been described to date.

The aim of the present study was to develop a protocol for the histological evaluation of immunostained hair cell synapses of cleared cochleae with the electrode array in situ, allowing evaluation of CI and its surrounding structures with a focus on synapses in one sample. Since new studies show an impact of noise on the ribbon synapses of the outer hair cells as well [33] both, inner and outer hair cell synapses, are in the focus of our developed method. By this, the here reported whole mount imaging method is usable for a wide range of research applications.

## 2. Materials and Methods

### 2.1. Animals

The staining was established on cochleae of an in-house breeding colony of albino and pigmented guinea pigs kindly provided by the working group of Prof. Mazzouli-Weber, Department of Physiology, University of Veterinary Medicine Hannover, Foundation, Hannover, Germany. Those guinea pigs were humanely killed by bolt shot in combination with throat transection, as permitted by the local authorities (§4) within the framework of the German “Law on Protecting Animals” and the cochleae were subsequently harvested.

Having established the staining protocol using those guinea pigs, a second set of cochleae were used to investigate if the protocol is feasible for intracardially perfused and fixed tissue as well. For this purpose Hartley guinea pigs from Charles River Laboratories, France were used. Euthanasia was performed by transcardial perfusion under general anesthesia (intramuscular medetomidinhydrochloride 0.2 mg/kg, midazolam 1 mg/kg and fentanyl 0.025 mg/kg) and additional local anesthesia with prilocain subcutaneously. The animals were perfused with 200 mL phosphate buffered saline, Sigma-Aldrich, (PBS) followed by 4% paraformaldehyde, Sigma-Aldrich. After decapitation the temporal bones were removed and dissected to expose the cochlea. This procedure was conducted in accordance with the German “Law on Protecting Animals” and with the European Communities Council Directive 2010/63/EU for the protection of animals used for experimental purposes. The use of animals for scientific purposes was permitted by the local authorities (Lower Saxony State Office for Consumer Protection and Food Safety (LAVES), Oldenburg, Germany, registration number 19/3145). In total the cochleae of 8 animals were used for this work.

### 2.2. Tissue Preparation

**Figure 1:**
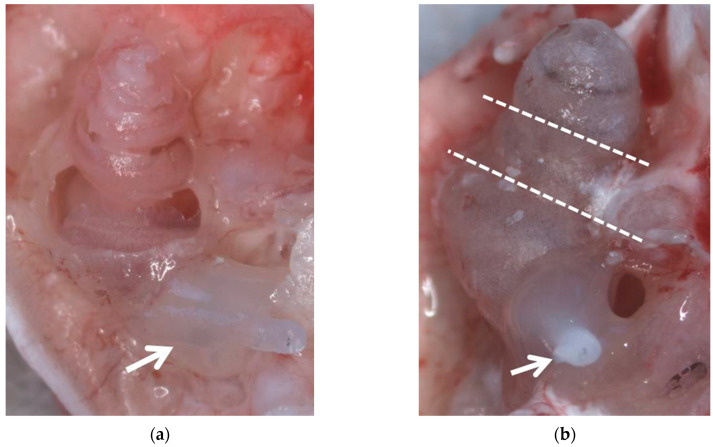
Both preparation methods after fixation with 4% PFA before decalcification. (**a**) Method II with dissected bony capsule. (**b**) Method III, the dotted lines indicate positions where the cochlea will be cut after decalcification. Arrows indicate the implants which are adhered to the round window niche using Tetric EvoFlow^®^.

**Figure 2:**
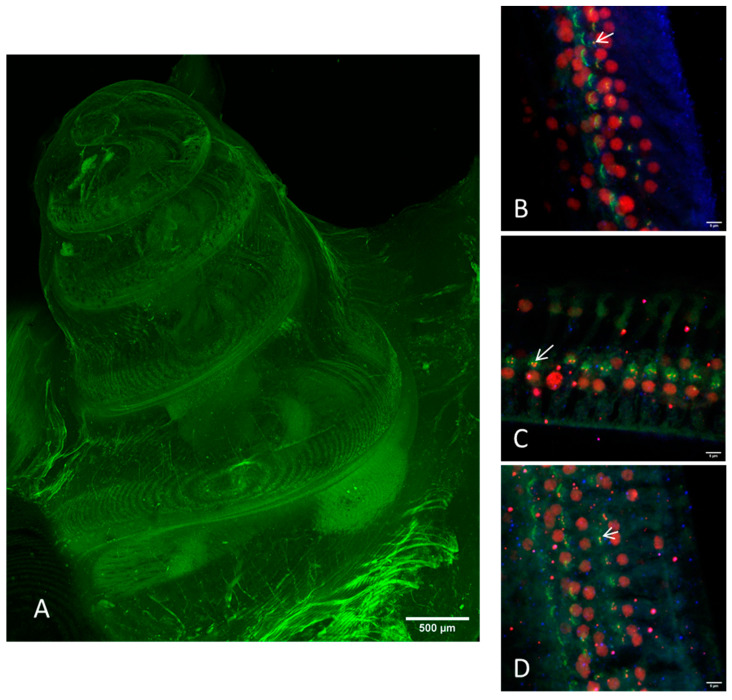
Images of the cleared intact guinea pig cochlea (method I) taken by CLSM. (**A**) Overview image with green autofluorescence using a 10× objective. Scale bar = 500 μm. (**B**–**D**) Immunostaining of the hair cells (myosin VIIa, blue), the presynaptic ribbon (CtBP2, red), and the postsynaptic density (PSD95, green). All images were acquired with a 20× objective on different positions: (**B**) apical, (**C**) median, (**D**) basal. Arrows indicate intact ribbon synapses, where the staining of the presynaptic ribbon and the one of the postsynaptic density are overlapping.

A small hole was drilled at the apex and the stapes was removed. The cochlea was slowly perfused with 4% paraformaldehyde (PFA) via the round window followed by 1 h fixation in 4% PFA on ice. A CI (kindly provided by MED-EL, Innsbruck, Austria) was inserted via the round window three to five mm into the scala tympani of the basal turn and fixed at the round window niche using Tetric Evo Flow^®^. Three different tissue preparation methods were investigated (Figure 1 and Figure 2): I. The cochlea stays intact (Figure 2); II. the bony shell of the cochlea was carefully removed with fine forceps starting at the apical hole (Figure 1a), leaving the modiolus and the lateral wall tissue intact by scratching with one shank of the forceps as close as possible to the bone; III the intact cochlea was decalcified and afterwards cut into a basal, medial, and apical part. All following steps were performed with gentle agitation at room temperature. After rinsing 3 × 10 min with phosphate buffered saline (PBS), the specimens were decalcified in 10% ethylenediamine tetraacetic acid-disodium salt (EDTA, Sigma-Aldrich) in PBS, pH 7.4, with EDTA changes every 1–3 days. After 21 days in EDTA the cochleae were washed 3 × 10 min with PBS. The intact cochleae of group III were dissected with a scalpel blade no. 11 into three parts horizontally to the modiolar axis (Figure 1b). Since the electrode array is located in the basal turn, it did not affect the cutting process.

### 2.3. Immunostaining

Immunostaining of ribbon synapses is possible using antibodies against the C-terminal binding protein 2 (CtBP2) and the postsynaptic density protein 95 (PDS95) [8,10,34]. CtBP2 is one of the two major domains of RIBEYE which is a specific component of synaptic ribbons [35]. PSD95 is a part of postsynaptic densities of the peripheral dendrites of the spiral ganglion neurons. If the fluorescence of both antibodies is overlapping, it is assumed that the synapse is intact [36,37]. The synapse-staining protocol is a modification of the one published by Shi et al. [34]. Additionally tissue was stained for myosin VIIa, which is located in the hair cells.

Permeabilization was achieved by 1% Triton-X (Sigma-Aldrich) in PBS for 45 min. The tissue was placed for 30 min in 50 mM ammonium chloride followed by 30 min blocking solution (20% horse serum/PBS). The incubation time of 21 h for the primary antibodies (mouse anti PSD95, 1:200, Merck MAB1596; mouse anti CtBP2, 1:250, BD #612044; rabbit anti myosin VIIa 1:250, Novus Biol. NB 120-3481) was followed by 3 × 10 min washing steps with PBS. Secondary antibodies (goat anti mouse IgG2a-Alexa488, 1:1000, Thermo Fisher #21131; goat anti mouse IgG1-Alexa 568, 1:1000, Thermo Fisher #21124; goat anti rabbit IgG-Cy5 1:500, Jackson #111-175-144) for 2 h followed by three washing steps (Table 1). Dehydration was achieved with ethanol and clearing with Spalteholz solution, consisting of five parts methyl salicylate and three parts benzyl benzoate (MSBB): 70% ethanol 3 h, 95% ethanol 30 min, absolute ethanol 2 h, absolute ethanol overnight, MSBB/ethanol (1:1) 4 h, MSBB 2 h, MSBB overnight.

### 2.4. Confocal Laser Scanning Microscopy (CLSM)

For imaging, the cochleae were placed in self-made glass chambers filled with MSBB as previously described [29]. The specimens of Method I and II were placed with the modiolar axis parallel to the glass sheet of the chamber and the apex was orientated downwards to reduce the distance to the objective lens. The dissected parts of Method III were placed with the cutted surface horizontally to the glass sheet. A Leica TCS SP8 confocal laser scanning microscope was used. CLSM of the whole mount cochlea for receiving overview images was performed using a 10×-objective (HC PL FLUOTAR 10×/0.30 DRY, Fa. Leica) and a 20×-objective (HC PL APO CS2 20×/0.75 IMM, Fa. Leica) as previously described in detail [29,30].

To investigate the pre- and postsynaptic structures the 20× objective (see above), i.e., 200-fold magnification, and a 63×-objective (HC CL APO CS2 63×/1.40 OIL, Fa. Leica), i.e., 630-fold magnification, with immersion oil (Leica Microsystems #11513859) were used. An additional optical zoom of about 1 was added for overview images and of about 7 was added for synapse imaging. Excitation was carried out using an argon-laser with 488 nm (Alexa 488), 568 nm (Alexa 568), and 650 nm (Cy5) (Table 1) absorption maxima and the photomultiplier gates were adjusted to 504–572 nm, 582–644 nm, and 654–783 nm, respectively. Stacks were generated in 1 µm steps for synapse overview images and in 0.3 µm steps for quantification of the synapses with a scanning speed of 600 Hz. The z-stacks had a final size of 9 to 45 μm. Following the previously published protocol, the images were exported and further processed using ImageJ software [30].

## 3. Results

With the established method for CLSM of the intact guinea pig cochlea based on the green autofluorescence [29] it was possible to generate a good overview image (Figure 2A). Imaging of the hair cell ribbon synapses, however, was nearly impossible. Imaging of the subcellular level was only possible in a few samples, but in a way that a reliable evaluation of the synapse number was not practicable (Figure 2B–D). Using the 20×-objective the distance to the synaptic structures is too long, because they are located too deep in the tissue. A higher magnification was impossible due to the limited working distance of the objectives in combination with the bony capsule. This implies that the plane of the ribbon synapses could not be placed in the focus plane of the 63×-objective.

**Figure 3:**
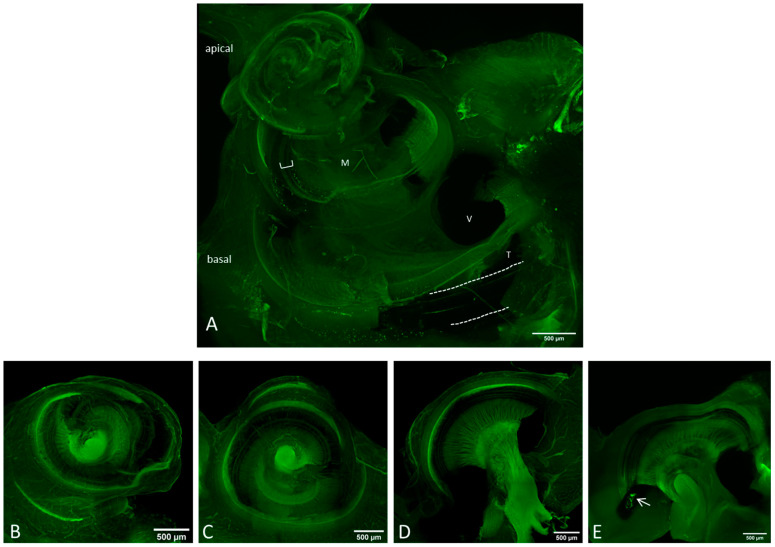
Overview images of the preparation methods II (**A**) and III (**B**–**E**) using a 10× objective with green autofluorescence. Scale bar = 500 μm. (**A**) The bony capsule was dissected. The lateral wall tissue is partially disrupted, but the organ of corti (bracket) seems to be intact. Dotted lines indicate the CI. M = modiolus, V = scala vestibule, T = scala tympani. (**B**) Apical, (**C**) middle and (**D**,**E**) basal part of the dissected cochlea. The arrow in (**E**) points on the CI.

**Figure 4:**
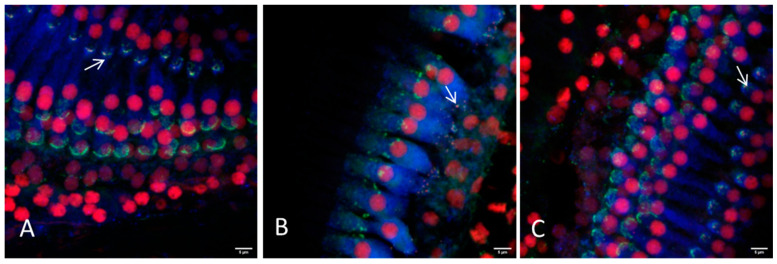

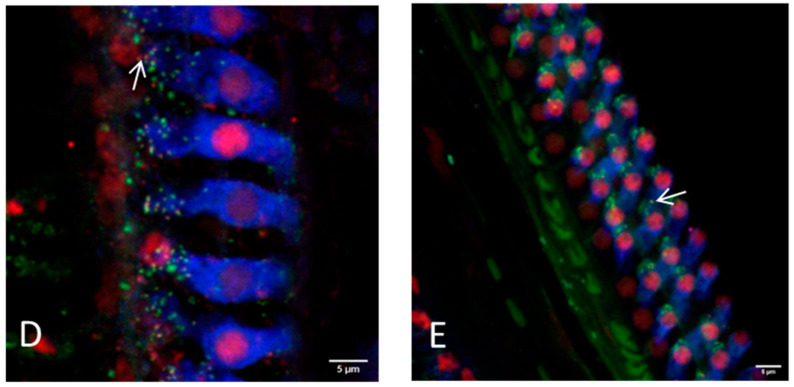
Representative images of the hair cell ribbon synapses of inner and outer hair cells with preparation method II (bony capsule dissected) using a 20× or a 63× objective. (**A**) Picture showing the apical region, where a distinction between inner and outer hair cells is not always possible due to overlapping of the cells. (**B**) Picture showing the inner hair cells and (**C**) the outer hair cells of the middle turn. (**D**,**E**) Inner and outer hair cells of the basal region are illustrated, respectively. Hair cells are labeled for myosin VIIa (blue), presynaptic ribbons for CtBP2 (red) and the postsynaptic density is labeled green. Arrows exemplarily indicate intact ribbon synapses, where the staining of the presynaptic ribbon and the postsynaptic density are overlapping. Scale bars indicate 5 μm.

**Figure 5:**
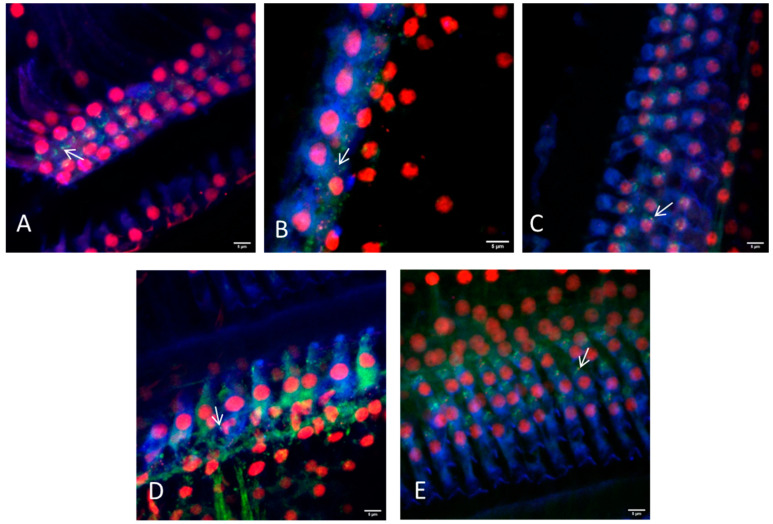
Representative images of the hair cell ribbon synapses of inner and outer hair cells with preparation method III (dissection in three parts) using a 20× or a 63× objective. (**A**) Picture showing the apical region, where a distinction between inner and outer hair cells is not always possible due to overlapping of the cells. (**B**) Picture showing the inner hair cells and (**C**) the outer hair cells of the middle turn. (**D**,**E**) Inner and outer hair cells of the basal region are illustrated, respectively. Hair cells are labeled for myosin VIIa (blue), presynaptic ribbons for CtBP2 (red), and the postsynaptic density is labeled green. Arrows indicate intact ribbon synapses, where the staining of the presynaptic ribbon and the postsynaptic density are overlapping. Scale bars indicate 5 μm.

**Figure 6:**
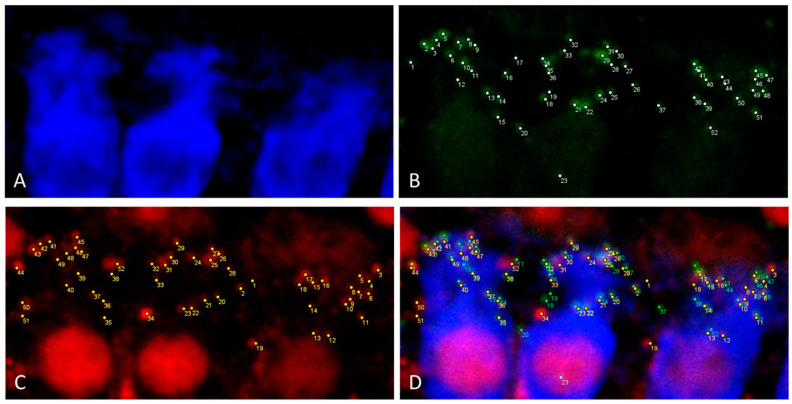
Illustration of the synapse quantification of three inner hair cells in maximal projection of a 0.3 μm z-stack using ImageJ. (**A**) Myosin VIIa (blue) labelling of inner hair cells. (**B**) Green PSD95 labelling of postsynaptic density. Every white point indicates a counted PSD95 staining. (**C**) Red CtBP2 labelling of presynaptic ribbon. Every yellow point indicates a counted CtBP2 staining. (**D**) Merging of the Pictures (**A**–**C**).

Based on this finding cochleae with removed bony capsule or with intact capsule but cut into three sections where investigated for their suitability for electrode array position determination in the overview images (Figure 3) and for synaptic imaging in higher resolution (Figure 4 and Figure 5). Using a 10×-objective overview images of the implanted cochlea with dissected bony capsule were possible to visualize and the electrode array was visualized in situ as well. Due to the preparation method the membranous tissue may in parts be disrupted (Figure 3A). Overview images of cochleae with intact capsule but cut into three sections (Figure 3B–E) provided information about the electrode position as well (Figure 3E). Using a 20× and sometimes a 63× objective (depending on the working distance between the sample and the objective) imaging of the ribbon synapses was possible with both preparation methods (Figure 4 and Figure 5). This way the visibility of the cells near to the CI depends on the sample positioning. The visualization of the co-localization of the presynaptic CtBP2 as part of the RIBEYE and the postsynaptic PSD95 was possible in the basal, middle, and apical parts of the implanted cochleae on inner and outer hair cells. Additionally a quantification of the synapses is possible using ImageJ (Figure 6). For this purpose z-stacks with a size of 0.3 µm were used. After determining a start and an end position for the scan the generation of the stacks for each sample position ran automatically, but with every scan the fluorescence intensity is reduced due to photobleaching.

The time for microscopy for cochleae with dissected capsules was shorter. The time commitment depended on the stability of the sample positioning. With the aforementioned method III it was more difficult to fixate the three parts in the glass chamber with MSBB, leading to a longer microscopy time. In return the time for preparation of method III is shorter than the one of method II and the tissue damage was only limited. Dissection of the bony capsule needs training and routine, but nevertheless a huge tissue damage especially of the lateral wall could be seen.

All preparation methods have their advantages and disadvantages (see Table 2 for more details).

Interestingly, a difference was found in the fluorescence intensity between pigmented and unpigmented guinea pigs, with albinos showing a higher intensity of staining for the antibodies used.

## 4. Discussion

Cleared cochleae are a good tool to visualize the complex three-dimensional structure of the inner ear. Unstained specimens can be investigated due to their autofluorescence [29]. Even implanted specimens can be imaged without big circumstances [30]. Using CLSM to image implanted cochleae, the presence of the CI itself affects the imaging. In some cases the implant induces a scattered radiation of the laser beam, leading to higher fluorescence in some areas of the tissue. This does not affect the synapse imaging. Next to this it is impossible to image through the CI which hinders the imaging of tissue that is behind the implant. The visibility of the cells near to the CI depends therefore on the sample positioning. Immunostaining can help to investigate specific components such as the inner hair cells, labelled with antibodies against otoferlin [31], spiral ganglion neurons, labelled with anti-neurofilament antibodies [25,26,31,38], fibrosis with vimentin-labelling [30] or IBA1 labelling for macrophages [32,38]. In this study the specimen was stained against the hair cell ribbon synapses using antibodies against CtBP2 and PSD95. As seen in other studies [34] the red fluorescence is not only present in the synapse region, but the nucleus is also stained. It originates from the fact that CtBP2 is not specific for ribbons, but the genes of RIBEYE, a component of the ribbon synapse, and CtBP2 have homologous sequences. This way ribbon synapses can be stained with antibodies against CtBP2 as well as CtBP2 itself, which is located in the nucleus [35]. The green fluorescence, indicating PSD 95 sometimes appeared as a non-specific labelling in other regions than the synapse level. The target structure of this phenomenon is not identified up to now. Sometimes this semicircular shaped immunolabelling appears at the synapse level, having no clear puncta but more elongated PSD95 staining and can therefore be distinguished from the postsynaptic density. Maybe the use of other postsynaptic markers such as GluA2 [8,37,39,40] is an option to circumvent this in future studies.

Nevertheless whole mount acquisition of the cochlea is very difficult due to several reasons [26]: the long acquisition time can lead to photobleaching, which is enhanced by light diffraction. The scanning process used in the current study was automated, but could only be repeated a few times due to the aforementioned effect of photobleaching. Another problem while working with large samples such as the intact guinea pig cochlea in confocal laser scanning microscopes is the working distance. Although the refractive index of 1.552 of the MSBB leads to a deeper penetration into the tissue it is still not possible with 200-fold or higher magnifications to reach the level of the ribbon synapses if the whole cochlea remains intact. The positioning of the specimen is not trivial because the bony capsule curvatures usually only allows a small number of positions. In contrast to other clearing methods the MSBB cleared specimen cannot be embedded in other materials. If the tissue is moved out of the liquid, the clearing process reverses. The clearing with iDISCO+ (a protocol based on methanol series, dichloromethane and dibenzylether) circumvents the problem of inaccurate sample positioning by embedding the tissue in gelatin, which can be cut to optimize the sample positioning [32]. In 100-fold magnification, the position of the sample is basically not a problem. However, the best possible position of the cochlea must be selected in this magnification, because this will make it easier to find the region of interest in the 200-fold magnification. Working with the entire cochlea, the bony capsule lies on the bottom of the glass chamber. To image the hair cells, the thickness of the bony capsule has first to be overcome. This reduces the number of measuring points because the working distance of the lens is limited. Measurements of the synapses in 630-fold magnification are not possible in intact cleared cochleae using our confocal laser scanning microscope. To solve this problem two preparation methods were compared, carefully removing the bony cochlea capsule before decalcification and cutting the cochlea horizontally to the axis in three sections after decalcification. With both methods we were able to visualize the ribbon synapses (Figure 4 and Figure 5), but every method had its advantages and disadvantages (Table 2). Both methods enabled ribbon synapse counting per hair cell (Figure 6) and can be applied to assess the effect of a therapy such as an implant or a delivered drug. To consider the three-dimensional arrangement of the synapses at the hair cell base, it seems useful to count the synapses manually while scrolling through the stack. This way no synapse should be missed. Additionally, when the position of the cochlea during the scanning process is documented, a statement of the approximate tonotopic location of the hair cells displayed by each z-stack is possible.

The removal of the bony capsule keeps the complex helical shape completely intact. This results in a good overview of all parts of the cochlea. In addition, false insertions of the implant into the vestibular system, which can occur by implanting via the round window, or basilar membrane perforation due to movement of the array from the scala tympani into the scala vestibule, can be noticed directly after harvesting of the temporal bones. Unfortunately, the removal of the bone is very difficult because the lateral wall structures are strongly attached to it. This way the lateral wall tissue can be disrupted accidently. Since the specimens of in vivo research are highly valuable, intensive training is needed before the tissue preparation is performed in a way that the membranous structures are always reliably preserved. Using a 100-fold magnification an overview image is quickly created because the sample is nearly always positioned in a way that the working distance allows deep imaging. The sample is usually stable in several positions (i.e., not moving when the objective is changed), so the transition to 200-fold magnification is easy. By removing the bony capsule, the regions of interest are exposed and can directly be imaged. There are a large number of measuring points because the cochlea can be rotated easily and remains stable in several positions. Hair cells are easy to find because they are close to the laser beam source. Inappropriate tissue regions due to preparation artifacts can be compensated for by the high number of measuring points. The restrictions due to the working distance of the lens are negligible in the 200-fold magnification. Using the 630-fold magnification the restrictions of the working distance are similar to that of an entire cochlea in the 200-fold magnification. Measurements in 630-fold magnification are possible.

Since the decalcification process itself has no significant impact to the sensory cells, it is a commonly used method for cochlear preparations. Cutting the cochlea into three sections after decalcification results in tissue damage. Due to the soft structure after decalcification the tissue is compressed while cutting, so the overlapping of the cells is intensified. A benefit of this method to cut the tissue is that the hair cells can be positioned directly on the glass surface of the imaging chamber. The amount of possible measurement points varies massively between specimens due to the described preparation damage which affects the Organ of Corti as well. Measurements of 630-fold magnification are possible.

The method of humanely killing the animal and therefore using tissue, which was either perfused over blood path, followed by perfusion via scala tympani or only via scala tympani, before one hour immersion in PFA did not have an impact on the immunohistochemical outcome. There was, however, a difference in fluorescence intensity between pigmented and unpigmented animals with pigmented guinea pigs having a less intense fluorescence. It is unclear whether the binding of the antibodies is reduced in pigmented animals or if the melanin impedes the detection of the fluorescence. To our knowledge this difference has not been reported before and it needs to be examined in future studies, because unpigmented as well as pigmented animals are used for research purposes.

Since the working distance of our microscope is the main limiting factor for imaging of the cochlea synapses, other imaging techniques may be applicable. One option for whole cochlea imaging is light sheet fluorescence microscopy (LSFM) [38,41,42] which also requires optical clearing of the tissue. This method as well offers the opinion to leave an implant in situ to evaluate both the amount of fibrosis and the volumetric degree of hair cell loss [38]. LSFM’s advantage over CLSM is the light efficiency, so the effect of photobleaching is reduced [43]. Nevertheless conventional LSFM reaches its limitations by visualizing the cellular level. Hutson et al. had to rehydrate and dissect the cochleae for visualization of the subcellular level with CLSM after LSFM, increasing the work load due to additional tissue preparation [42,44]. Meanwhile a new method of LSFM is developed called cleared tissue axially swept light-sheet microscopy (ctASLM) [45], which achieves subcellular resolution in cleared tissues. Moreover there are several studies working on the subcellular resolution of light-sheet microscopy [46,47,48], but they only tested specimens smaller than the guinea pig cochlea without an implant. The development of new imaging techniques especially for cleared tissue is a contemporary issue. Maybe the new generation of light sheet microscopes, which is adapted to a wide span of refractive indices of different clearing methods, enables imaging of larger samples as the guinea pig cochlea with resolution at subcellular level. This has to be investigated in future studies.

Today CLSM is the method of choice, because it gives the opportunity to image the implanted cochlea to evaluate the implant position and the tissue reaction onto the implantation, the sensory cells, and spiral ganglion neurons. Our study proves for the first time that the first synapses of the auditory system can also be imaged in whole mount-implanted cochleae.

## 5. Conclusions

We developed a technique to visualize the ribbon synapses of cleared guinea pig cochleae with a CI in situ. Removal of the bony wall or dissection into three parts allow the evaluation of the CI position as well as visualization of the sensory cells including the synapses. If the aim is to evaluate the components of the Organ of Corti and therefore damage to the lateral wall tissue is negligible, the removal of the bony capsule of the cochlea is superior over three-pieces dissection. Additionally, eventually damaged regions of the basilar membrane due to preparation artefacts can be compensated by the high number of possible measuring points, so a safe evaluation is given. This novel technique can be applied in a wide field of hearing research focusing on synaptopathy.

## Figures and Tables

**Table 1:** Combination of the primary and secondary antibodies, with their target structure and the emission color.

Target Structure	Primary Antibody	Secondary Antibody	Excitation Wavelength (nm)	Emission Color
Hair cell	Rabbit anti myosin VIIa, Novus Biol. NB 120-3481	Goat anti rabbit IgG-Cy5, Jackson #111-175-144	650	Blue
Postsynaptic density	Mouse anti PSD95, Merck MAB1596	Goat anti mouse IgG2a-Alexa488, Thermo Fisher #21131	488	Green
Ribbon synapse	Mouse anti CtBP2, BD #612044	Goat anti mouse IgG1-Alexa568, Thermo Fisher #21124	568	Red

**Table 2:** Comparison of the different preparation methods concerning preparation, imaging and tissue integrity. Symbols stand for “+++” optimal, “++” good, “+” acceptable, “+/−” challenging, “−” nearly impossible.

	I—Intact Cochlea	II—Bony Capsule Dissected	III—Dissected in Three Sections
Preparation time	+++	+	++
Time for microscopy	+	+++	++
20× objective	+	++	+++
63× objective	−	+	+
Stable sample positioning	++	++	+/−
Basilar membrane damage due to preparation	+++	+/−	+
Overview image	+++	+	++
Synapse imaging	−	+	++
Lateral wall tissue	intact	Often disrupted	Mostly intact

## Data Availability

The data presented in this study are available on request from the corresponding author.
